# Lipoprofiling Assessed by NMR Spectroscopy in Patients with Acute Coronary Syndromes: Is There a Need for Fasting Prior to Sampling?

**DOI:** 10.3390/diagnostics12071675

**Published:** 2022-07-10

**Authors:** Laura-Adina Stănciulescu, Alexandru Scafa, Cătălin Duduianu, Raluca Stan, Alina Nicolescu, Calin Deleanu, Maria Dorobanțu

**Affiliations:** 1 Department of Cardiology, Emergency Clinical Hospital, 014461 Bucharest, Romania; laurastanciulescu95@gmail.com (L.-A.S.); alexscafa@yahoo.com (A.S.); 2 Faculty of Medicine, “Carol Davila” University of Medicine and Pharmacy, 050513 Bucharest, Romania; maria.dorobantu@gmail.com; 3 “C.D. Nenitescu” Centre of Organic Chemistry, Romanian Academy, 060023 Bucharest, Romania; duduxian94@gmail.com; 4 Faculty of Applied Chemistry and Material Science, University Politehnica of Bucharest, 011061 Bucharest, Romania; raluca.stan@upb.ro; 5 “Petru Poni” Institute of Macromolecular Chemistry, Romanian Academy, 700487 Iasi, Romania

**Keywords:** dyslipidemia, cardiovascular disease, acute coronary syndrome, NMR spectroscopy, lipidomics, metabolomics, lipoprotein subclasses, fasting, statin

## Abstract

Most patients presenting in an emergency unit with acute coronary syndromes (ACS) (which include non-ST-elevation myocardial infarction (NSTEMI), ST-elevation MI (STEMI), and unstable angina) usually meet at least two cardiovascular risk factors, such as dyslipidemia, arterial hypertension, diabetes mellitus type 2, obesity, history of or current smoking, etc. Most ACS patients suffer from a type of dyslipidemia, and in addition to this there are ACS patients rushed into the emergency units for which the feeding status is unknown. Thus, we set out to evaluate the effect of fasting on 16 blood metabolite concentrations and 114 lipoprotein parameters on one control group and a group of statin-treated ACS patients hospitalized in a cardiovascular emergency unit, using Nuclear Magnetic Resonance (NMR) spectroscopy. The results indicated trends (in terms of number of cases, but not necessarily in terms of the magnitude of the effect) for as many as four metabolites and 48 lipoproteins. The effect was defined as a trend for results showing over 70% of the cases from either one or both groups that experienced parameter changes in the same direction (i.e., either increased or decreased). In terms of magnitude, the effect is rather low, leading to the overall conclusion that in cardiovascular (CV) emergency units, the blood samples analyzed in any feeding status would provide close results and very valuable information regarding prognosis and for fast decisions on patient’s proper management.

## 1. Introduction

Cardiovascular diseases (CVDs) are one of the main causes of death and morbidity in the world at the moment. Both women and men have high rates of cardiovascular morbi-mortality, even though gender-related differences in mortality and morbidity rates are observed in different populational age groups. Differences associated with sex are observed in the clinical course and manifestations, which raises the suspicion that gender influences processes related to atherosclerosis. Atherosclerotic cardiovascular disease (ACD) includes two main clinical manifestations: ischemic heart disease (which summarizes ACS–IHD) and cerebrovascular disease (mainly ischemic stroke) [1].

Ischemic heart disease (IHD) and stroke are the most common causes of death in the population, with 84.5% of deaths associated with CVDs and 28.2% of all-cause mortality [2,3,4].

The three main types of ACS include non-ST-elevation myocardial infarction (NSTEMI), ST-elevation MI (STEMI), and unstable angina.

Most patients presenting in an emergency unit with ACS usually meet at least two cardiovascular risk factors, such as dyslipidaemia, arterial hypertension, diabetes mellitus type 2, obesity, history of or current smoking, etc. [5]. Of all the major risk factors, dyslipidemia remains one of the most important in the assessment of the cardiovascular high-risk patients. Therefore, lipoprofiling, even in emergency units, is of high importance.

The current practice on biochemical analysis of blood is to sample it in fasting status. There are, however, recent reports questioning the need for fasting prior to sampling at least for a selected set of biochemical parameters, but the only such study that covered both fasting status and CV events examined only the main lipoprotein parameters [6].

In emergency cardiovascular units, where life-threatening cases are being frequently managed, it is important to assess the plasmatic LDL and cholesterol levels. However, when a serious cardiovascular event occurs, the fasting or non-fasting status of the patient is often unknown. Nevertheless, even in non-emergency situations, some patients are hiding their failure to fast before sampling.

Based on the above considerations, we set out the objective to assess the effect of fasting on lipoprotein profiling in cardiovascular emergency units, by selecting a random population of 29 patients admitted with ACS, either with previous or newly introduced statin treatment, and a control group of 24 healthy individuals, who have been subjected to advanced lipoprofiling by NMR spectroscopy.

## 2. Materials and Methods

### 2.1. Group Selection

The CVD group included 29 subjects (ages between 38 and 78 years, 17 males, 15 smokers, 14 diabetic, 12 hypertensive, 17 suffering from dyslipidaemia), with acute coronary syndromes (ACS) hospitalized in a tertiary cardiovascular emergency unit at the time of the evaluation. Some of them were already on statin treatment prior to their admittance, while others were newly introduced on this treatment. All subjects received 80 mg atorvastatin for lipid-lowering the evening before the blood sampling.

The control group included 24 healthy individuals (ages between 26 and 59 years, 5 males, 5 smokers), without any known comorbidities and without any long-term treatment at the moment of sampling. One diabetic case and two other subjects that have been diagnosed with high LDL and triglyceride concentrations during this study have been excluded from the statistics, and they are not among the 24 selected controls.

Out of the total of 53 subjects, 12 subjects performed the same test on two or three different days, thus leading to a total of 68 pairs of fasting/postprandial experiments (cases), out of which there were 29 controls and 39 CVD.

### 2.2. Blood Sampling

A blood sample of 6 mL was collected before and two hours after meals in red capped sterile plain tubes (standardized red vacutainers without any anticoagulant, used for regular serological examination in biochemistry) and centrifuged after 30 min at room temperature at 3500 rpm for 15 min for serum separation. The separated serum was subsequently aliquoted in 1 mL cryovials and stored at −80 °C until NMR analysis.

### 2.3. NMR Analysis

The cryovials were kept at −80 °C until up to 60 min before the NMR analysis. Before analysis, the cryovials were allowed to defrost at room temperature for 20 to 30 min. The sample consisting of 400 µL plasma and 400 µL of 5 mM sodium 3-(trimethylsilyl)-[2,2,3,3-d4]-1-propionate (TSP) in Na_2_HPO4/NaN_3_/D_2_O buffer (Bruker Biospin, Ettlingen, Germany) was homogenized at room temperature with a plasma rotator (VWR). A volume of 600 µL of the homogenized sample was then transferred into a 5 mm NMR tube (Wilmad 507) and further loaded into the NMR sample changer. The operator’s performance and the NMR tubes’ quality were assessed as we previously described [7]. The NMR experiments were performed with an Avance III HD 600 NMR spectrometer (Bruker Biospin, Ettlingen) operating at 600.12 MHz for ^1^H nuclei, equipped with a 5 mm inverse detection (BBI) probe with gradients on the *z*-axis. Five different types of NMR experiments have been recorded at 300.0 K with pulse sequences and parameters as delivered with Bruker Biospin IVDr methods V.1.0 [8], i.e., ^1^H NMR spectrum with an ERETIC signal as quantitation reference, J-Resolved 2D spectrum for signal assignments, 1D Diffusion filtered spectrum for suppression of low molecular weight metabolites, CPMG spectrum for suppression of lipids, and ^1^H Gradient profile for quality control of the sample preparation (sample homogeneity and tube quality). A set of 3 Bruker QC standard samples have been recorded before each series of plasma samples, in order to assess (and further correct if necessary) the compliance of the instrument with temperature stability (±0.05 K), water suppression quality, and ERETIC quantitation accuracy (±2%). We have used the commercial B.I.LISA methods and models (Bruker Biospin, Ettlingen) to analyze the IVDr raw NMR data [8,9].

The full set of assessed metabolites included: alanine (Ala), creatinine (Crn), glutamine (Glut), glycine (Gly), histidine (His), isoleucine (i-Leu), phenylalanine (Phe), tyrosine (Tyr), valine (Val), acetic acid (Ac), formic acid (For), lactic acid (Lac), pyruvic acid (Pyr), and glucose (Gluc).

The full set of lipoprotein parameters included **Lipoprotein Main Fractions**: ***Total Lipids and lipoproteins*** (Total Triglycerides (TPTG), Total Cholesterol (TPCH), Total Apo-A1 (TPA1), Total Apo-A2 (TPA2), Total Apo-B100 (TPAB), ApoB100/ApoA1 (ABA1), Total Particle number (TPPN)), ***High-Density Lipoproteins*** (HDL-Apo-A1 (HDA1), HDL-Apo-A2 (HDA2), HDL-Cholesterol (HDCH), HDL-Free cholesterol (HDFC), HDL-Phospholipids (HDPL), HDL-Triglycerides (HDTG)), ***Low-Density Lipoproteins*** (LDL-Apo-B100 (LDAB), LDL-Cholesterol (LDCH), LDL-Free cholesterol (LDFC), LDL-Phospholipids (LDPL), LDL-Particle number (LDPN), LDL-Triglycerides (LDTG), LDL-chol/HDL-chol ratio (LDHD)), ***Intermediate-Density Lipoproteins*** (IDL-Apo-B100 (IDAB), IDL-Cholesterol (IDCH), IDL-Free cholesterol (IDFC), IDL-Phospholipids (IDPL), IDL-Particle number (IDPN), IDL-Triglycerides (IDTG)), ***Very Low-Density Lipoproteins*** (VLDL-Apo-B100 (VLAB), VLDL-Cholesterol (VLCH), VLDL-Free cholesterol (VLFC), VLDL-Phospholipids (VLPL), VLDL-Particle number (VLPN), VLDL-Triglycerides (VLTG)). **Lipoprotein Subfractions** included: ***High-Density Lipoproteins(HDL1-4)***: High-Density Lipoprotein 1 (HDL1-Apo-A1 (H1A1), HDL1-Apo-A2 (H1A2), HDL1-Cholesterol (H1CH), HDL1-Free cholesterol (H1FC), HDL1-Phospholipids (H1PL), HDL1-Triglycerides (H1TG)), High-Density Lipoprotein 2 (HDL2-Apo-A1 (H2A1), HDL2-Apo-A2 (H2A2), HDL2-Cholesterol (H2CH), HDL2-Free cholesterol (H2FC), HDL2-Phospholipids (H2PL), HDL2-Triglycerides (H2TG)), High-Density Lipoprotein 3 (HDL3-Apo-A1 (H3A1), HDL3-Apo-A2 (H3A2), HDL3-Cholesterol (H3CH), HDL3-Free cholesterol (H3FC), HDL3-Phospholipids (H3PL), HDL3-Triglycerides (H3TG)), High-Density Lipoprotein 4 (HDL4-Apo-A1 (H4A1), HDL4-Apo-A2 (H4A2), HDL4-Cholesterol (H4CH), HDL4-Free cholesterol (H4FC), HDL4-Phospholipids (H4PL), HDL4-Triglycerides (H4TG)), ***Low-Density Lipoproteins (LDL1-6)***: Low-Density Lipoprotein 1 (LDL1-ApoB100 (L1AB), LDL1-Cholesterol (L1CH), LDL1-Free cholesterol (L1FC), LDL1-Phospholipids (L1PL), LDL1-Particle number (L1PN), LDL1-Triglycerides (L1TG)), Low-Density Lipoprotein 2 (LDL2-ApoB100 (L2AB), LDL2-Cholesterol (L2CH), LDL2-Free cholesterol (L2FC), LDL2-Phospholipids (L2PL), LDL2-Particle number (L2PN), LDL2-Triglycerides (L2TG)), Low-Density Lipoprotein 3 (LDL3-ApoB100 (L3AB), LDL3-Cholesterol (L3CH), LDL3-Free cholesterol (L3FC), LDL3-Phospholipids (L3PL), LDL3-Particle number (L3PN), LDL3-Triglycerides (L3TG)), Low-Density Lipoprotein 4 (LDL4-ApoB100 (L4AB), LDL4-Cholesterol (L4CH), LDL4-Free cholesterol (L4FC), LDL4-Phospholipids (L4PL), LDL4-Particle number (L4PN), LDL4-Triglycerides (L4TG)), Low-Density Lipoprotein 5 (LDL5-ApoB100 (L5AB), LDL5-Cholesterol (L5CH), LDL5-Free cholesterol (L5FC), LDL5-Phospholipids (L5PL), LDL5-Particle number (L5PN), LDL5-Triglycerides (L5TG)), Low-Density Lipoprotein 6 (LDL6-ApoB100 (L6AB), LDL6-Cholesterol (L6CH), LDL6-Free cholesterol (L6FC), LDL6-Phospholipids (L6PL), LDL6-Particle number (L6PN), LDL6-Triglycerides (L6TG)), ***Very Low-Density Lipoproteins (VLDL1-5)***: Very Low-Density Lipoprotein 1 (VLDL1-Cholesterol (V1CH), VLDL1-Free cholesterol (V1FC), VLDL1-Phospholipids (V1PL), VLDL1-Triglycerides (V1TG)), Very Low-Density Lipoprotein 2 (VLDL2-Cholesterol (V2CH), VLDL2-Free cholesterol (V2FC), VLDL2-Phospholipids (V2PL), VLDL2-Triglycerides (V2TG)), Very Low-Density Lipoprotein 3 (VLDL3-Cholesterol (V3CH), VLDL3-Free cholesterol (V3FC), VLDL3-Phospholipids (V3PL), VLDL3-Triglycerides (V3TG)), Very Low-Density Lipoprotein 4 (VLDL4-Cholesterol (V4CH), VLDL4-Free cholesterol (V4FC), VLDL4-Phospholipids (V4PL), VLDL4-Triglycerides (V4TG)), Very Low-Density Lipoprotein 5 (VLDL5-Cholesterol (V5CH), VLDL5-Free cholesterol (V5FC), VLDL5-Phospholipids (V5PL), VLDL5-Triglycerides (V5TG)).

## 3. Results

We evaluated the effect of fasting on 16 blood metabolite concentrations and 114 lipoprotein parameters on one control group and one group of statin-treated patients hospitalized in a cardiovascular emergency unit, using NMR spectroscopy.

Statin administration for lipid-lowering was performed the evening before the blood sampling. It is well known that endogenous cholesterol synthesis is cyclical in nature with the greatest production during fasting states, and therefore statins, especially the ones with short half-life, should be administered in the evening, thus allowing the greatest drug concentration to be present during peak endogenous cholesterol synthesis. Statin is the gold standard for dyslipidemia treatment [10].

The food intake effect was assessed as trends based on the number of similar changes in parameters for each pair of fasting/non-fasting samples from the same subject.

In order to enhance the food intake effect and to downgrade the influence of other factors, given the small pilot group of subjects, the results have been processed as percentage change in parameters for each pair of fasting/non-fasting samples from the same subject (case). Thus, it is assumed that the general status of the organism does not change significantly in a matter of a few hours, and the observed effect could be safely assigned to feeding. When the same subject performed the test in more than one day, the percentages of each pair was considered as a separate case (datapoint) and the results were not averaged for the same subject. On the contrary, for 56 out of the total of 68 tests, more than one serum vial was analyzed separately, and the results of these multiple analyses were averaged in order to improve the analytical accuracy. The accuracy and reproducibility of NMR metabolomics and lipidomics has been recently demonstrated by large trials [8,9,11,12], and we have also described recent reproducibility assessments for the NMR method [7,13]. A subset of samples was checked for some parameters (total cholesterol, total triglycerides, total LDL, and total HDL) through both NMR standardized methods of the hospital’s biochemistry laboratory, and the two methods are in good agreement (Supplementary Table S2).

A large panel of blood metabolites and lipoprotein parameters have been assessed by NMR spectroscopy. Trends have been considered when over 70% of the cases from either one or both groups experienced parameter changes in the same direction. Thus, significative changes have been observed for four metabolites (glycine, leucine, phenylalanine, lactate) (Table 1) and 48 lipoproteins parameters (TPTG, VLPN, L5PN, L6PN, VLTG, IDTG, HDTG, VLCH, IDCH, VLFC, LDFC, VLPL, IDPL, LDPL, VLAB, V1TG, V2TG, V3TG, V1CH, V2CH, V1FC, V2FC, V5FC, V1PL, V2PL, V3PL, L2TG, L2CH, L5CH, L6CH, L1FC, L4FC, L5FC, L2PL, L6PL, L5AB, L6AB, H1TG, H2TG, H3TG, H3CH, H4CH, H1FC, H2FC, H4FC, H4PL, H4A1, H4A2) (Table 2).

Interestingly, for one low-density free cholesterol subfraction (L1FC) the concentration trends for the two groups were in opposite directions (i.e., decreased and increased concentrations after meals for the control and CVD groups, respectively). For the four metabolites (Gly, Leu, Phal, Lac) and five lipoprotein parameters (L6PN, L2TG, L6CH, L6PL, L6AB), only the CVD group exhibited a quantifiable trend, while for 27 lipoprotein parameters (HDTG, VLCH, IDCH, VLFC, LDFC, LDPL, V1TG, V1CH, V2CH, V1FC, V2FC, V5FC, V1PL, V2CH, L4FC, L2PL, H1TG, H2TG, H3TG, H3CH, H4CH, H1FC, H2FC, H4FC, H4PL, H4A1, H4A2) only the control group exhibited a quantifiable trend. There were 15 lipoprotein parameters for which both groups exhibited similar trends (TPTG, VLPN, L5PN, VLTG, IDTG, VLPL, IDPL, VLAB, V2TG, V3TG, V2PL, V3PL, L5CH, L5FC, L5AB), and as much as 12 metabolites and 66 lipoprotein parameters for which none of the two group exhibited any quantifiable trend.

The results for low molecular weight blood metabolites are presented in Table 1. The concentration for each pair of fasting-postprandial samples is defined as percentage difference F-P considering the fasting (F) concentration as 100%. The number of positive and negative pairs (cases) is expressed as percentage from the total number of pairs (cases) in each group.

Similarly, the results for lipoprotein-related parameters are presented in Table 2.

**Figure 1:**
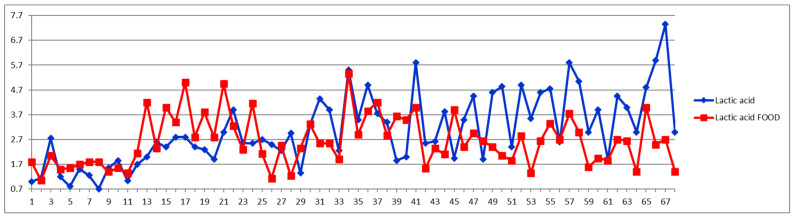
Lactic acid concentrations (mmol/L) in fasting (blue) and postprandial (red) status for control cases (1–29) and CVD cases (30–68).

The small metabolites evaluation is exemplified below for lactic acid (Lac), which, as shown in Table 1, exhibits a quantifiable trend for CVD group after food intake. Figure 1 shows two Shewhart charts with individual experiments marked in blue for fasting and in red for postprandial samples. Each number on the *x* axis represents a case, with numbers 1–29 belonging to controls and 30–68 belonging to CVD patients. The *y* axis represents the Lac concentration in mmol/L. One can see concentrations ranging between 0.7–7.3 mmol/L depending on the individual organism. As a general trend, one can conclude that most of the Lac concentrations are higher after food intake for most of controls (red curve above for cases 1–29) and lower after food intake for almost all CVD patients (red curve below for cases 30–68).

**Figure 2:**
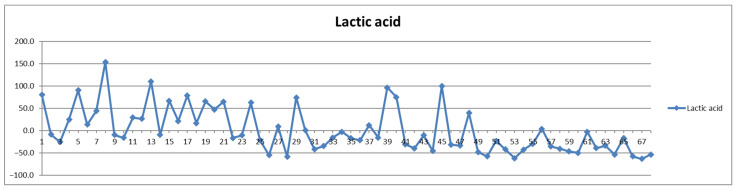
The food intake effect on lactic acid metabolism as difference between postprandial and fasting concentrations as percentage relative to fasting concentration.

The second way of processing the results was as differences of concentrations between postprandial and fasting cases. Thus, in the Shewhart chart presented in Figure 2, each point represents the corresponding concentration difference between the postprandial (red) and fasting (blue) as a percentage relative to fasting status (considered 100%). One can see the general trend of the curve above 0% for the control group (cases 1–29) and below 0% for the CVD group (cases 30–68) representing increased and decreased Lac concentration after food intake for the two groups, respectively.

**Figure 3:**
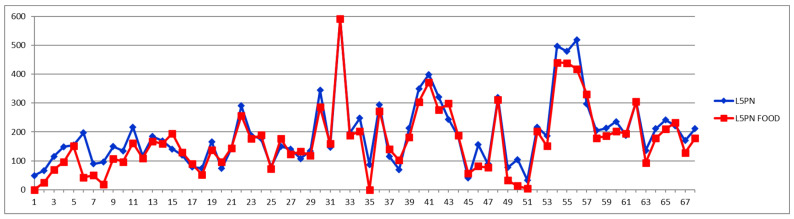
Small LDL particle number (nmol/L) subfraction L5PN in fasting (blue) and postprandial (red) status for control cases (1–29) and CVD cases (30–68).

**Figure 4:**
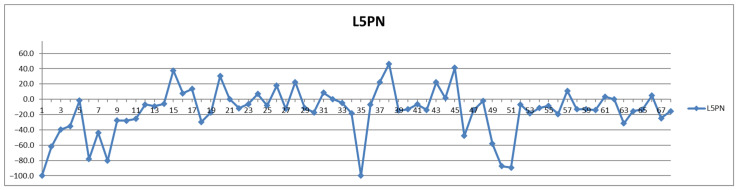
The food intake effect on small LDL particle number L5PN subfraction as difference between postprandial and fasting concentrations as percentage relative to fasting concentration.

The same processing is exemplified in Figure 3 and Figure 4 for the particle number of small-dense Low-Density Lipoproteins (s-LDL) particle number subfraction L5PN. As it can be seen from Table 2, in over 73% of all cases (from both controls and CVD) these fractions are decreased postprandial, which is evident from Figure 3 and Figure 4, i.e., the red curve below the blue one in most of the cases and, respectively, the different curve below zero in most of the cases.

Currently, the LDL particle number is proven to be more reliable for estimating cardiovascular risk than LDL cholesterol [14,15], with small-dense LDL subfractions (L5-6PN) being considered the best predictors of CV incidents. In this paradigm, the question of whether the feed induced diminishing of these subfractions could lower the warning flag of future CV incidents is becoming important.

As it can be seen from Figure 4, the postprandial diminishing of L5PN is less than 30% in most cases, although in four cases it is diminished by 80% and in two cases even to zero or undetectable levels (−100%).

**Figure 5:**
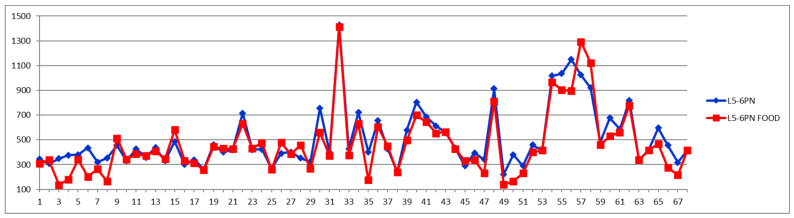
Small LDL particle number (L5PN+L6PN) (nmol/L) in fasting (blue) and postprandial (red) status for control cases (1–29) and CVD cases (30–68).

Although the border level of small-dense LDL particle number (L5PN + L6PN) for high CV risk is still under discussion, in general it is considered that a level between 100–600 nmol/L is of medium risk, while above 600 nmol/L is of high risk and above 900 nmol/L is of very high risk for CV accidents. Thus, lowering an already low level of s-LDL-PN is of no diagnosis significance. The question is if lowering a high-level s-LDL-PN could mislead the course of intervention in emergency CV units. For this, we examined the feeding effect on the total levels of s-LDL particle numbers, i.e., L5PN + L6PN (Figure 5). One observation from the Shewhart chart presented in Figure 5 is that with five exceptions (i.e., one CVD subject with cases 57 and 58 from different days, and three control cases 15, 26, 28) in all the other 63 cases, the s-LDL-PN concentrations are either diminished or almost unaffected by feeding. These five increased postprandial cases should be treated as exceptions, probably due to particular foods and/or very light meals. Out of the 63 cases for which the feeding is diminishing the s-LDL-PN level, there is only one case for which the feeding effect is misleading the diagnosis by shifting the result from high-risk to low-risk (case 30); all the other cases remain in the same class of risk. Nevertheless, this finding is supporting the concern that for cases close to the border between either high-to-very high or low-to-high levels, there might be a feeding-induced change of risk class. The answer to this concern should be a classical approach, namely that in all border cases a second sample should be collected in the fasting state in order to safely decide on patient management.

**Figure 6:**
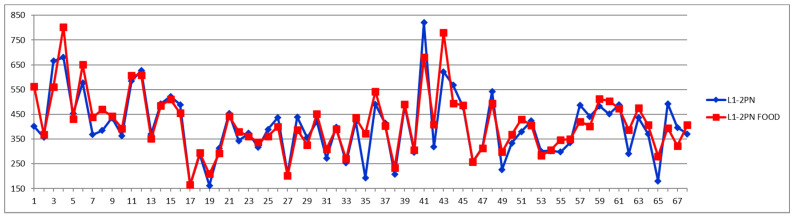
Large LDL particle number (L1PN + L2PN) (nmol/L) in fasting (blue) and postprandial (red) status for control cases (1–29) and CVD cases (30–68).

**Figure 7:**
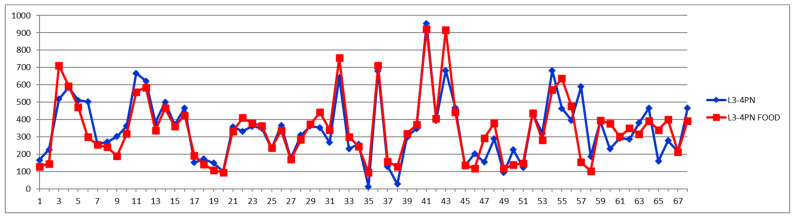
Medium LDL particle number (L3PN + L4PN) (nmol/L) in fasting (blue) and postprandial (red) status for control cases (1–29) and CVD cases (30–68).

**Figure 8:**
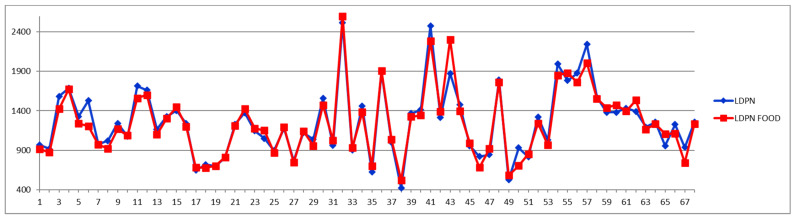
Total LDL particle number (LDPN) (nmol/L) in fasting (blue) and postprandial (red) status for control cases (1–29) and CVD cases (30–68).

For comparison, Figure 6, Figure 7 and Figure 8 show the feeding effect on large, medium, and total LDL-PN fractions, respectively.

When total LDL particles (LDPN) are considered for evaluating the CVD risk, the following levels are often used: low—(<1000 nmol/L), moderate—(1000–1300 nmol/L), borderline-high—(1300–1600 nmol/L), high—(1600–2000 nmol/L), and very high-risk—(>2000 nmol/L).

A comparison of Figure 5, Figure 6, Figure 7 and Figure 8 confirms the modern paradigm that LDL is not the best predictor for CV accidents and that the real status could be masked by balanced concentrations of large versus small LDL subfractions. Thus, we may highlight three types of situations exemplified by cases 32, 41, and 54. Looking to the total LDL particle number (Figure 8), one can conclude that the three cases are in a similarly bad prognosis, with cases 32 and 41 in the very high-risk group with LDL-P well above 2000 nmol/L and the case 54 being at the border of high- to very high-risk groups. However, looking to the distribution of large (L1PN + L2PN) and medium LDL (L3PN + L4PN) versus small-dense LDL (L5PN + L6PN) in Figure 5, Figure 6 and Figure 7, respectively, one would reach a different assessment. Thus, due to unbalanced low level of large LDL-P (Figure 6) and high levels of medium (Figure 7) and small LDL-P (Figure 5), the case 32 is assigned to the same high-risk group; however, due to the same unbalanced distribution of LDL particles, case 41 is in a much better situation, based on the small LDL-P (Figure 5) being assigned to the border between medium and high risk. Based on the small LDL-P particles, case 54 is assigned to the very high-risk group but close to the high-risk border and in a much better situation than case 32.

In terms of feeding-induced effects, one can see that even if the general trend is in most of the cases a decrease of small LDL particles (Figure 5), the overall pattern for all cases remains the same and the effect is relatively small, not significantly affecting the diagnosis and patient management. Of course, when cases are at the border of risk levels, one could have some difficulties in assigning the course of action (e.g., case 30 in Figure 5).

**Figure 9:**
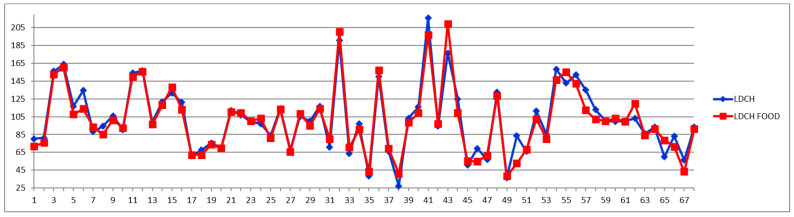
LDL cholesterol (LDCH) concentrations (mg/dL) in fasting (blue) and postprandial (red) status for control cases (1–29) and CVD cases (30–68).

**Figure 10:**
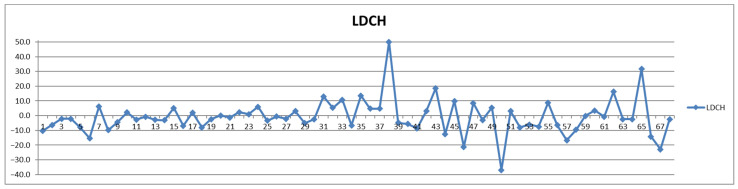
The food intake effect on LDL cholesterol (LDCH) as difference between postprandial and fasting concentrations as percentage relative to fasting concentration.

**Figure 11:**
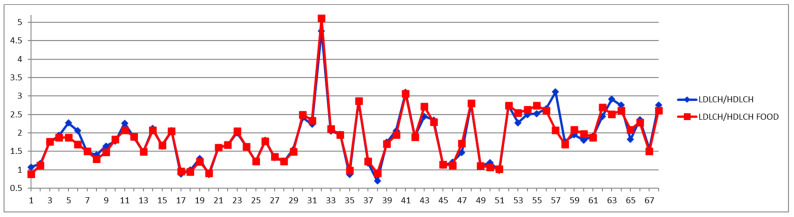
LDL/HDL cholesterol ratios in fasting and postprandial status in fasting (blue) and postprandial (red) status for control cases (1–29) and CVD cases (30–68).

**Figure 12:**
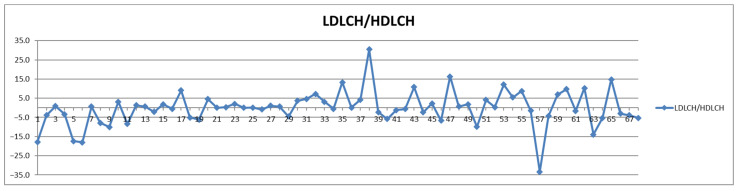
The food intake effect on LDL/HDL cholesterol ratios as difference between postprandial and fasting concentrations as percentage relative to fasting concentration.

On the other hand, if one stays with the classical approach, namely considering the total LDL particle numbers (Figure 8), as well as the total LDL cholesterol (Figure 9 and Figure 10) and the LDL-C/HDL-C ratios (Figure 11 and Figure 12), we can conclude that with very few exceptions, for this approach the feeding status is not influencing the diagnosis. As this classical approach is still a widespread approach in CVD management, both due to the lack of access to state-of-the-art NMR diagnosis tools and due to much larger reference databases published for these parameters, we may conclude that the feeding status makes no difference for this approach. Thus, if one considers the food intake effect on LDL cholesterol (LDCH), there is no feed-induced trend (Table 2), and in fact the individual variations for most of the cases are well within ±20% (Figure 10), comparable with daily/weekly variations and not affecting the diagnosis. The same is true with the effect of the food intake on the LDL cholesterol to HDL cholesterol ratios (Figure 11 and Figure 12).

**Figure 13:**
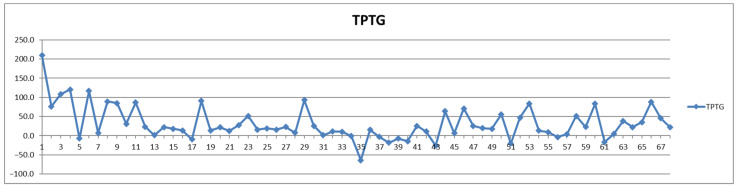
The food intake effect on total triglycerides (TPTG) as difference between postprandial and fasting concentrations as percentage relative to fasting concentration.

**Figure 14:**
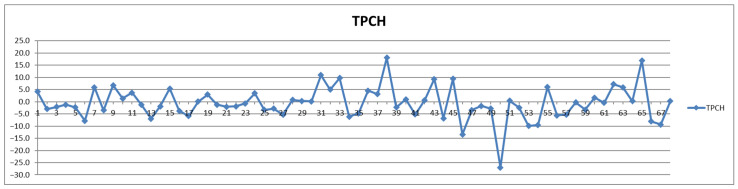
The food intake effect on total cholesterol (TPCH) as difference between postprandial and fasting concentrations as percentage relative to fasting concentration.

**Figure 15:**
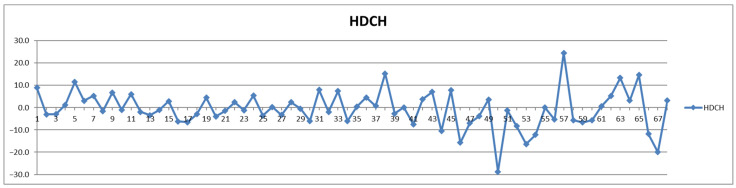
The food intake effect on HDL cholesterol (HDCH) as difference between postprandial and fasting concentrations as percentage relative to fasting concentration.

**Figure 16:**
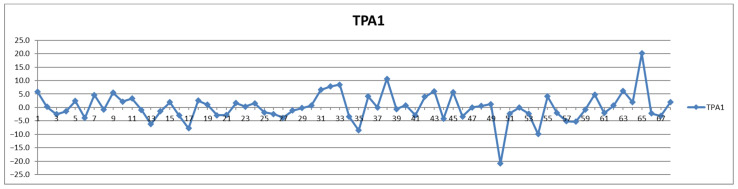
The food intake effect on total apolipoprotein A1 (TPA1) as difference between postprandial and fasting concentrations as percentage relative to fasting concentration.

**Figure 17:**
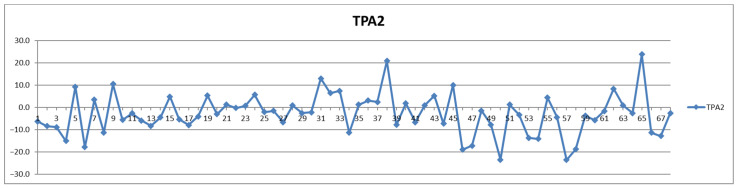
The food intake effect on total apolipoprotein A2 (TPA2) as difference between postprandial and fasting concentrations as percentage relative to fasting concentration.

**Figure 18:**
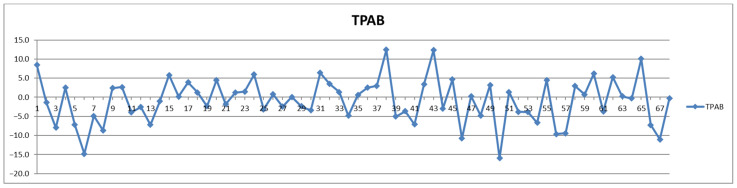
The food intake effect on total apolipoproteins B100 (TPAB) as difference between postprandial and fasting concentrations as percentage relative to fasting concentration.

**Figure 19:**
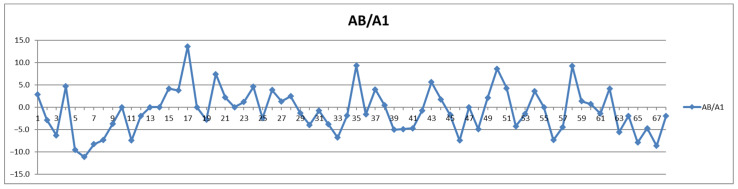
The food intake effect on Apo-B100/Apo-A1 (ABA1) as difference between postprandial and fasting concentrations as percentage relative to fasting concentration.

In addition to the parameters discussed above, the management of cardiovascular patients also relies on values for total triglycerides (TPTG), total cholesterol (TPCH), HDL cholesterol (HDCH), total apolipoproteins A1 (TPA1), total apolipoproteins A2 (TPA2), total apolipoproteins B100 (TPAB), and Apo-B100/Apo-A1 (ABA1). Out of these parameters, only total triglycerides (TPTG) exhibit a sizable feeding-induced trend, i.e., with postprandial levels increased in over 80% of cases (Table 2). On the other hand, with the exception of TPTG for which in several cases the feeding induces increases of over 50% (Figure 13), for all the other parameters the feeding effect is in almost all cases well within ±20% (Figure 14, Figure 15, Figure 16, Figure 17, Figure 18 and Figure 19).

The complete set of NMR results for the parameters listed in Table 1 and Table 2 for all 68 individual cases in both fasting and postprandial status is presented as Supplementary Information in the excel Table S1.

## 4. Discussion

In this research, we set out to assess whether there is a certain pattern of lipid variation before and after a meal in ACS patients admitted in an emergency cardiovascular unit. We assessed the influence of food intake on a large dataset of small molecular weight metabolites and lipoprotein parameters by NMR spectroscopy.

Overall, we noticed that, besides the obvious and well-known increase in triglycerides after food intake in both the CVD and the control group, there were no significant changes before and after food intake, although for several parameters a tendency pattern could be identified. Thus, feeding-induced trends have been observed for four metabolites (glycine, leucine, phenylalanine, lactate) and 48 lipoproteins parameters (TPTG, VLPN, L5PN, L6PN, VLTG, IDTG, HDTG, VLCH, IDCH, VLFC, LDFC, VLPL, IDPL, LDPL, VLAB, V1TG, V2TG, V3TG, V1CH, V2CH, V1FC, V2FC, V5FC, V1PL, V2PL, V3PL, L2TG, L2CH, L5CH, L6CH, L1FC, L4FC, L5FC, L2PL, L6PL, L5AB, L6AB, H1TG, H2TG, H3TG, H3CH, H4CH, H1FC, H2FC, H4FC, H4PL, H4A1, H4A2).

For one parameter, feeding induced opposite trends for the two groups while for some other cases there was a clear tendency for only one of the two groups. For the case when feeding induced different trends in the two groups, the explanation could be a combination of the specific and homogenous hospital diet, as well as the treatment effect, most notably the statin administration for all CVD cases.

ACS patients are regarded as high-risk patients for developing several major cardiovascular events. Some of them are already known to have dyslipidemia, while others are newly diagnosed upon admission. Risk factor control is of great importance in these patients, and the effectiveness of their lipid-lowering treatment is usually measured by serum levels of LDL-cholesterol (LDL-C) [16]. Currently, the LDL particle number is proven to be more reliable for estimating cardiovascular risk than LDL cholesterol, with small-dense LDL subfractions (L5-6PN) being considered the best CV incidents predictors. We noticed a decrease in L5PN levels after food intake, usually within 30%, although in several cases it decreased by 80% and in two cases even to zero or undetectable levels (−100%). We examined the effect of food intake on the total levels of L5PN + L6PN, and we noticed that in the majority of cases, the s-LDL-PN concentration is either diminished or almost unaffected by food intake.

Many studies have evaluated the association between LDL subfractions and cardiovascular outcomes. However, as a systematic review has shown, few of these were performed with an identical measurement method, namely NMR, and different cut-points were used for the various LDL subfractions. Despite limitations, the review concluded that LDL particle number was associated with incident cardiovascular disease, while LDL particle size and small LDL particle fraction were not as much [17]. A recent review concludes that small dense subfraction of LDL is more indicative for CHD risk prediction than total LDL [18].

Another recently performed study based on patients with coronary artery disease (CAD), compared to a control group, showed that triglycerides, LDL-C3, LDL-C4, LDL-C5, LDL-C6, and total LDL-C levels were significantly higher in patients with CAD, while LDL-C1 and HDL-C were significantly lower in the same group. The same review points out that lipid parameters associations with ischemic stroke in patients with non-valvular atrial fibrillation are still contradictory [19]. Therefore, we agree that sampling LDL-C subfractions on larger populational groups would help create better prediction scores for major CV events in the general population.

Although many studies using non-fasting blood samples for assessment of lipid levels found that decreasing levels of non-fasting lipids reduces the risk of CV disease, there is still no sound scientific evidence up to this date as to why fasting is superior to non-fasting when evaluating a lipid profile in order to assess and predict the cardiovascular risk. However, non-fasting samples have many advantages: they simplify the blood sampling in the laboratory, they do not impose the need for fasting, therefore there is no need for the patient to have their blood drawn early in the morning, and, moreover, for individuals with diabetes, the risk of hyperglycemia due to fasting would be minimized [20].

Overall, our NMR study assessed that, with the exception of total triglycerides, all the other classical parameters used in the management of cardiovascular patients (total cholesterol, LDL cholesterol, HDL cholesterol, LDL-C/HDL-C ratios, total apolipoproteins A1, total apolipoproteins A2, total apolipoproteins B100, and total Apo-B100/Apo-A1), as well as other 66 lipoprotein parameters, including several subclasses, exhibit no specific trend and are not significantly affected by the fasting status. This result is in agreement with other recent studies [4,20]. On the other hand, we assessed that four metabolites and 48 lipoprotein parameters, including total triglycerides, and modern markers used in CVD management (small-dense subfractions of LDL particle numbers) show a postprandial-induced trend in over 70% of cases. These results also confirm feeding-induced effects recently reported on healthy volunteers [21,22,23,24,25], although we observed opposite trends for the CVD group in comparison with controls for one parameter, and for 36 parameters out of the 130 studied parameters, only one of the two groups exhibited a quantifiable food-induced trend. On the other hand, our NMR study assessed that, with the exception of triglycerides, the magnitude of the feeding effect is relatively small and could have an effect on CVD management only for some borderline cases. Thus, the combined assessment of several CVD markers which can be obtained in the same NMR analysis would safely allow the patient management in CV emergency units in any fasting status.

Although we are assessing that the magnitude of the feeding effect would not affect the CVD patients’ management in emergency units, the detectable feeding effect on 52 parameters and particularly the opposite trend in CVD versus controls for one parameter as well as the identification of nine parameters for which a feeding-induced trend is observed only in CVD but not in controls, could bring valuable insights in the mechanisms of the CVD as well as in therapy response assessment.

There are, however, a series of limitations to this pilot study. First of all, the research was performed on a relatively small number of subjects and the conclusions cannot be extended to the general population. Secondly, the age range does not overlap completely. Thirdly, even though both groups followed the same study protocol, not all of them were fed the same meal, mostly because the patients were not admitted at the same time, therefore it would have been impossible to have the exact same meal.

Therefore, further studies in larger cohorts are needed to confirm our results and to draw firm conclusions. In order to facilitate comparison with future studies, all our row data are presented in Supplementary Table S2. With atherosclerosis becoming one of the main causes or morbi-mortality worldwide, we strongly believe it is important to further assess the relationship between a therapeutic intervention and how different changes in lipoprotein subfractions impact risk for major cardiovascular events.

## 5. Conclusions

There were no significant changes before and after food intake in both groups regarding most tested parameters, with the exception of triglycerides, where increased values were observed after food intake in both groups. Thus, even though feeding-induced trends have been observed for several parameters, with the exception of triglycerides, the magnitude of the effects should not affect the decision on CVD patients’ management in emergency units.

Our findings strengthen the idea that the old paradigm of imposing fasting prior to blood sampling for CVD assessment purposes is no longer valid. Thus, at least for emergency situations, the NMR lipoprofiling is a powerful tool for prognosis and in CVD management decisions, regardless of the patient’s fasting status.

Small-dense LDL subfractions (L5-6PN) have recently been considered the best predictors for CV major events. We noticed a decrease in L5PN levels after food intake, usually within 40%. However, further studies, ideally on larger populational cohorts, should be performed in order to assess the association between LDL subfractions and cardiovascular outcomes in non-fasting states and to create risk prediction scores with conclusions that can be extended to the general population.

## Figures and Tables

**Table 1:** Trends for small metabolites evolution after food intake. Significant changes (marked in bold) are considered when more than 70% of cases exhibit the same trend.

Metabolite	Total				Control				Cardio			
	% >0	% <0	% =0	Cases	% >0	% <0	% =0	Cases	% >0	% <0	% =0	Cases
Ala	47.1	47.1	5.9	68	62.1	31.0	6.9	29	35.9	59.0	5.1	39
Crn	39.7	52.9	7.4	68	31.0	55.2	13.8	29	46.2	51.3	2.6	39
Glut	52.9	47.1	0.0	68	51.7	48.3	0.0	29	53.8	46.2	0.0	39
Gly	33.8	63.2	2.9	68	58.6	37.9	3.4	29	15.4	**82.1**	2.6	39
His	42.6	48.5	8.8	68	51.7	31.0	17.2	29	35.9	61.5	2.6	39
i-Leu	44.1	45.6	10.3	68	69.0	27.6	3.4	29	25.6	59.0	15.4	39
Leu	27.9	**70.6**	1.5	68	48.3	48.3	3.4	29	12.8	**87.2**	0.0	39
Phal	29.4	57.4	13.2	68	41.4	37.9	20.7	29	20.5	**71.8**	7.7	39
Tyr	39.7	44.1	16.2	68	37.9	37.9	24.1	29	41.0	48.7	10.3	39
Val	36.8	57.4	5.9	68	34.5	62.1	3.4	29	38.5	53.8	7.7	39
Ac	42.6	38.2	19.1	68	48.3	34.5	17.2	29	38.5	41.0	20.5	39
Cit	52.9	42.6	4.4	68	65.5	27.6	6.9	29	43.6	53.8	2.6	39
For	42.6	36.8	20.6	68	34.5	48.3	17.2	29	48.7	28.2	23.1	39
Lac	38.2	61.8	0.0	68	65.5	34.5	0.0	29	17.9	**82.1**	0.0	39
Pyr	52.9	41.2	5.9	68	69.0	17.2	13.8	29	41.0	59.0	0.0	39
Gluc	52.9	44.1	2.9	68	44.8	55.2	0.0	29	59.0	35.9	5.1	39

**Table 2:** Trends for lipoprotein evolution after food intake. Significant changes (marked in bold) are considered when more than 70% of cases exhibit the same trend.

Lipo-Fraction	Total				Control				Cardio			
	% >0	% <0	% =0	Cases	% >0	% <0	% =0	Cases	% >0	% <0	% =0	Cases
TPTG	**82.4**	17.6	0.0	68	**93.1**	6.9	0.0	29	**74.4**	25.6	0.0	39
TPCH	44.1	55.9	0.0	68	37.9	62.1	0.0	29	48.7	51.3	0.0	39
LDCH	38.2	61.8	0.0	68	31.0	69.0	0.0	29	43.6	56.4	0.0	39
HDCH	45.6	52.9	1.5	68	44.8	55.2	0.0	29	46.2	51.3	2.6	39
TPA1	48.5	51.5	0.0	68	44.8	55.2	0.0	29	51.3	48.7	0.0	39
TPA2	36.8	63.2	0.0	68	31.0	69.0	0.0	29	41.0	59.0	0.0	39
TPAB	50.0	50.0	0.0	68	48.3	51.7	0.0	29	51.3	48.7	0.0	39
LDHD	51.5	45.6	2.9	68	44.8	48.3	6.9	29	56.4	43.6	0.0	39
ABA1	38.2	52.9	8.8	68	44.8	41.4	13.8	29	33.3	61.5	5.1	39
TBPN	50.0	50.0	0.0	68	48.3	51.7	0.0	29	51.3	48.7	0.0	39
VLPN	**73.5**	26.5	0.0	68	**75.9**	24.1	0.0	29	**71.8**	28.2	0.0	39
IDPN	44.1	55.9	0.0	68	41.4	58.6	0.0	29	46.2	53.8	0.0	39
LDPN	41.2	58.8	0.0	68	34.5	65.5	0.0	29	46.2	53.8	0.0	39
L1PN	48.5	51.5	0.0	68	31.0	69.0	0.0	29	61.5	38.5	0.0	39
L2PN	64.7	35.3	0.0	68	69.0	31.0	0.0	29	61.5	38.5	0.0	39
L3PN	54.4	45.6	0.0	68	37.9	62.1	0.0	29	66.7	33.3	0.0	39
L4PN	42.6	52.9	4.4	68	31.0	65.5	3.4	29	51.3	43.6	5.1	39
L5PN	26.5	**73.5**	0.0	68	27.6	**72.4**	0.0	29	25.6	**74.4**	0.0	39
L6PN	41.2	58.8	0.0	68	62.1	37.9	0.0	29	25.6	**74.4**	0.0	39
VLTG	**79.4**	20.6	0.0	68	**86.2**	13.8	0.0	29	**74.4**	25.6	0.0	39
IDTG	**82.4**	16.2	1.5	68	**89.7**	6.9	3.4	29	**76.9**	23.1	0.0	39
LDTG	66.2	33.8	0.0	68	62.1	37.9	0.0	29	69.2	30.8	0.0	39
HDTG	67.6	32.4	0.0	68	**79.3**	20.7	0.0	29	59.0	41.0	0.0	39
VLCH	69.1	30.9	0.0	68	**72.4**	27.6	0.0	29	66.7	33.3	0.0	39
IDCH	36.8	61.8	1.5	68	24.1	**72.4**	3.4	29	46.2	53.8	0.0	39
LDCH	38.2	61.8	0.0	68	31.0	**69.0**	0.0	29	43.6	56.4	0.0	39
HDCH	45.6	52.9	1.5	68	44.8	55.2	0.0	29	46.2	51.3	2.6	39
VLFC	**73.5**	25.0	1.5	68	**82.8**	13.8	3.4	29	66.7	33.3	0.0	39
IDFC	42.6	52.9	4.4	68	34.5	58.6	6.9	29	48.7	48.7	2.6	39
LDFC	41.2	58.8	0.0	68	27.6	**72.4**	0.0	29	51.3	48.7	0.0	39
HDFC	58.8	41.2	0.0	68	58.6	41.4	0.0	29	59.0	41.0	0.0	39
VLPL	**75.0**	25.0	0.0	68	**79.3**	20.7	0.0	29	**71.8**	28.2	0.0	39
IDPL	**77.9**	22.1	0.0	68	**79.3**	20.7	0.0	29	**76.9**	23.1	0.0	39
LDPL	41.2	58.8	0.0	68	27.6	**72.4**	0.0	29	51.3	48.7	0.0	39
HDPL	48.5	51.5	0.0	68	48.3	51.7	0.0	29	48.7	51.3	0.0	39
HDA1	42.6	57.4	0.0	68	41.4	58.6	0.0	29	43.6	56.4	0.0	39
HDA2	36.8	63.2	0.0	68	31.0	69.0	0.0	29	41.0	59.0	0.0	39
VLAB	**73.5**	26.5	0.0	68	**75.9**	24.1	0.0	29	**71.8**	28.2	0.0	39
IDAB	44.1	55.9	0.0	68	41.4	58.6	0.0	29	46.2	53.8	0.0	39
LDAB	41.2	58.8	0.0	68	34.5	65.5	0.0	29	46.2	53.8	0.0	39
V1TG	**73.5**	26.5	0.0	68	**86.2**	13.8	0.0	29	64.1	35.9	0.0	39
V2TG	**82.4**	17.6	0.0	68	**93.1**	6.9	0.0	29	**74.4**	25.6	0.0	39
V3TG	**73.5**	26.5	0.0	68	**75.9**	24.1	0.0	29	**71.8**	28.2	0.0	39
V4TG	60.3	39.7	0.0	68	51.7	48.3	0.0	29	66.7	33.3	0.0	39
V5TG	41.2	58.8	0.0	68	37.9	62.1	0.0	29	43.6	56.4	0.0	39
V1CH	**73.5**	25.0	1.5	68	**79.3**	17.2	3.4	29	69.2	30.8	0.0	39
V2CH	67.6	29.4	2.9	68	**72.4**	24.1	3.4	29	64.1	33.3	2.6	39
V3CH	57.4	41.2	1.5	68	62.1	34.5	3.4	29	53.8	46.2	0.0	39
V4CH	41.2	58.8	0.0	68	34.5	65.5	0.0	29	46.2	53.8	0.0	39
V5CH	44.1	55.9	0.0	68	51.7	48.3	0.0	29	38.5	61.5	0.0	39
V1FC	**75.0**	20.6	4.4	68	**86.2**	10.3	3.4	29	66.7	28.2	5.1	39
V2FC	**72.1**	23.5	4.4	68	**75.9**	13.8	10.3	29	69.2	30.8	0.0	39
V3FC	67.6	27.9	4.4	68	69.0	20.7	10.3	29	66.7	33.3	0.0	39
V4FC	42.6	52.9	4.4	68	37.9	51.7	10.3	29	46.2	53.8	0.0	39
V5FC	25.0	67.6	7.4	68	13.8	**72.4**	13.8	29	33.3	64.1	2.6	39
V1PL	**77.9**	22.1	0.0	68	**89.7**	10.3	0.0	29	69.2	30.8	0.0	39
V2PL	**79.4**	20.6	0.0	68	**89.7**	10.3	0.0	29	**71.8**	28.2	0.0	39
V3PL	**73.5**	26.5	0.0	68	**75.9**	24.1	0.0	29	**71.8**	28.2	0.0	39
V4PL	48.5	51.5	0.0	68	41.4	58.6	0.0	29	53.8	46.2	0.0	39
V5PL	45.6	52.9	1.5	68	44.8	51.7	3.4	29	46.2	53.8	0.0	39
L1TG	61.8	36.8	1.5	68	51.7	44.8	3.4	29	69.2	30.8	0.0	39
L2TG	**72.1**	27.9	0.0	68	65.5	34.5	0.0	29	**76.9**	23.1	0.0	39
L3TG	41.2	58.8	0.0	68	34.5	65.5	0.0	29	46.2	53.8	0.0	39
L4TG	51.5	47.1	1.5	68	41.4	55.2	3.4	29	59.0	41.0	0.0	39
L5TG	51.5	48.5	0.0	68	51.7	48.3	0.0	29	51.3	48.7	0.0	39
L6TG	39.7	60.3	0.0	68	51.7	48.3	0.0	29	30.8	69.2	0.0	39
L1CH	52.9	47.1	0.0	68	37.9	62.1	0.0	29	64.1	35.9	0.0	39
L2CH	**70.6**	29.4	0.0	68	**72.4**	27.6	0.0	29	69.2	30.8	0.0	39
L3CH	58.8	39.7	1.5	68	44.8	55.2	0.0	29	69.2	28.2	2.6	39
L4CH	38.2	54.4	7.4	68	31.0	65.5	3.4	29	43.6	46.2	10.3	39
L5CH	25.0	**75.0**	0.0	68	27.6	**72.4**	0.0	29	23.1	**76.9**	0.0	39
L6CH	35.3	64.7	0.0	68	51.7	48.3	0.0	29	23.1	**76.9**	0.0	39
L1FC	54.4	45.6	0.0	68	27.6	**72.4**	0.0	29	**74.4**	25.6	0.0	39
L2FC	66.2	33.8	0.0	68	69.0	31.0	0.0	29	64.1	35.9	0.0	39
L3FC	54.4	45.6	0.0	68	48.3	51.7	0.0	29	59.0	41.0	0.0	39
L4FC	41.2	58.8	0.0	68	27.6	**72.4**	0.0	29	51.3	48.7	0.0	39
L5FC	23.5	**76.5**	0.0	68	24.1	**75.9**	0.0	29	23.1	**76.9**	0.0	39
L6FC	42.6	57.4	0.0	68	55.2	44.8	0.0	29	33.3	66.7	0.0	39
L1PL	52.9	47.1	0.0	68	34.5	65.5	0.0	29	66.7	33.3	0.0	39
L2PL	69.1	30.9	0.0	68	**72.4**	27.6	0.0	29	66.7	33.3	0.0	39
L3PL	52.9	45.6	1.5	68	37.9	58.6	3.4	29	64.1	35.9	0.0	39
L4PL	45.6	52.9	1.5	68	31.0	69.0	0.0	29	56.4	41.0	2.6	39
L5PL	30.9	69.1	0.0	68	31.0	69.0	0.0	29	30.8	69.2	0.0	39
L6PL	30.9	69.1	0.0	68	44.8	55.2	0.0	29	20.5	**79.5**	0.0	39
L1AB	48.5	51.5	0.0	68	31.0	69.0	0.0	29	61.5	38.5	0.0	39
L2AB	64.7	35.3	0.0	68	69.0	31.0	0.0	29	61.5	38.5	0.0	39
L3AB	54.4	45.6	0.0	68	37.9	62.1	0.0	29	66.7	33.3	0.0	39
L4AB	42.6	52.9	4.4	68	31.0	65.5	3.4	29	51.3	43.6	5.1	39
L5AB	26.5	**73.5**	0.0	68	27.6	**72.4**	0.0	29	25.6	**74.4**	0.0	39
L6AB	41.2	58.8	0.0	68	62.1	37.9	0.0	29	25.6	**74.4**	0.0	39
H1TG	**72.1**	27.9	0.0	68	**79.3**	20.7	0.0	29	66.7	33.3	0.0	39
H2TG	60.3	39.7	0.0	68	**75.9**	24.1	0.0	29	48.7	51.3	0.0	39
H3TG	67.6	32.4	0.0	68	**72.4**	27.6	0.0	29	64.1	35.9	0.0	39
H4TG	69.1	30.9	0.0	68	69.0	31.0	0.0	29	69.2	30.8	0.0	39
H1CH	67.6	32.4	0.0	68	65.5	34.5	0.0	29	69.2	30.8	0.0	39
H2CH	55.9	44.1	0.0	68	65.5	34.5	0.0	29	48.7	51.3	0.0	39
H3CH	44.1	55.9	0.0	68	24.1	**75.9**	0.0	29	59.0	41.0	0.0	39
H4CH	30.9	69.1	0.0	68	24.1	**75.9**	0.0	29	35.9	64.1	0.0	39
H1FC	67.6	32.4	0.0	68	**72.4**	27.6	0.0	29	64.1	35.9	0.0	39
H2FC	38.2	61.8	0.0	68	20.7	**79.3**	0.0	29	51.3	48.7	0.0	39
H3FC	41.2	55.9	2.9	68	27.6	69.0	3.4	29	51.3	46.2	2.6	39
H4FC	35.3	63.2	1.5	68	17.2	**82.8**	0.0	29	48.7	48.7	2.6	39
H1PL	64.7	35.3	0.0	68	62.1	37.9	0.0	29	66.7	33.3	0.0	39
H2PL	61.8	38.2	0.0	68	62.1	37.9	0.0	29	61.5	38.5	0.0	39
H3PL	47.1	52.9	0.0	68	31.0	69.0	0.0	29	59.0	41.0	0.0	39
H4PL	35.3	64.7	0.0	68	17.2	**82.8**	0.0	29	48.7	51.3	0.0	39
H1A1	63.2	36.8	0.0	68	65.5	34.5	0.0	29	61.5	38.5	0.0	39
H2A1	51.5	48.5	0.0	68	48.3	51.7	0.0	29	53.8	46.2	0.0	39
H3A1	48.5	51.5	0.0	68	34.5	65.5	0.0	29	59.0	41.0	0.0	39
H4A1	29.4	**70.6**	0.0	68	17.2	**82.8**	0.0	29	38.5	61.5	0.0	39
H1A2	64.7	35.3	0.0	68	65.5	34.5	0.0	29	64.1	35.9	0.0	39
H2A2	64.7	35.3	0.0	68	58.6	41.4	0.0	29	69.2	30.8	0.0	39
H3A2	61.8	38.2	0.0	68	51.7	48.3	0.0	29	69.2	30.8	0.0	39
H4A2	27.9	**72.1**	0.0	68	24.1	**75.9**	0.0	29	30.8	69.2	0.0	39

## Data Availability

All data and experimental details were included in the manuscript and Supplementary Materials.

## Supplementary Materials

The following supporting information can be downloaded at: https://www.mdpi.com/article/10.3390/diagnostics12071675/s1, Supplementary-1: Lipoprotein subfraction densities and abbreviations; Supplementary-2—Table S1: NMR results; Supplementary-3—Table S2: Comparison between classical and NMR results.
