# Supplementary material for: Lipoprofiling Assessed by NMR Spectroscopy in Patients with Acute Coronary Syndromes: Is There a Need for Fasting Prior to Sampling?
Source: Diagnostics (Basel). 2022 Jul 10;12(7):1675. doi: 10.3390/diagnostics12071675 (PMC9319954; doi:10.3390/diagnostics12071675)
Supplement: Supplementary file 1 [file diagnostics-12-01675-s001.zip › Supplementary-1.pdf]

SUPPLEMENTARY INFORMATION FOR:

Lipoprofiling assed by NMR spectroscopy in cardiovascular emergency units. Is need for fasting prior to sampling?

Laura-Adina Stănciulescu, Alexandru Scafa, Cătălin Duduianu, Raluca Stan, Alina Nicolescu, Calin Deleanu and Maria Dorobanțu

Lipoprotein Main fractions and densities:

High Density Lipoprotein (HDL): 1.063 - 1.210 kg/L
Low Density Lipoprotein (LDL): 1.019 - 1.063 kg/L
Intermediate Density Lipoprotein (IDL): 1.006 - 1.019 kg/L
Very Low Density Lipoprotein (VLDL): 0.950 - 1.006 kg/L

High Density Lipoprotein Subfractions:

HDL-1: 1.063 - 1.100 kg/L
HDL-2: 1.100 - 1.112 kg/L
HDL-3: 1.112 - 1.125 kg/L
HDL-4: 1.125 - 1.210 kg/L

Low Density Lipoprotein Subfractions (LDL1-3: Large LDL; LDL4-6: Small Dense LDL):

LDL-1: 1.019 - 1.031 kg/L
LDL-2: 1.031 - 1.034 kg/L
LDL-3: 1.034 - 1.037 kg/L
LDL-4 1.037 - 1.040 kg/L
LDL-5: 1.040 - 1.044 kg/L
LDL-6: 1.044 - 1.063 kg/L

Very Low Density Lipoprotein Subfractions:

VLDL-1, VLDL-2, VLDL-3, VLDL-4, VLDL-5, numbering according to increasing density with subfractions properties are specified in [S1].

[S1] Lindgren FT, Jensen LL, Hatch FT (1972) The isolation and quantitative analysis of serum lipoproteins. In: Nelson GJ (ed.) Blood lipids and lipoproteins: Quantitation, composition and metabolism. Wiley-Interscience, New York, p 181-274.

Lipoprotein Abbreviations and measuring units:

Total Plasma/Serum	Triglycerides	TPTG	mg/dL
Total Plasma/Serum	Cholesterol	TPCH	mg/dL
Total Plasma/Serum	Free Cholesterol	TPFC	mg/dL
Total Plasma/Serum	Apo-A1	TPA1	mg/dL

Total Plasma/Serum Apo-A2 TPA2 mg/dL
Total Plasma/Serum Apo-B100 TPAB mg/dL
Total Plasma/Serum Particle Number TPPN nmol/L
Total Plasma/Serum Apo-B100/Apo-A1 ratio TBA1 -/-
Total Plasma/Serum LDL-Cho/HDL-Cho ratio LDHD -/-
VLDL Triglycerides mg/dL
VLDL Cholesterol VLCH mg/dL
VLDL Free Cholesterol VLFC mg/dL
VLDL Phospholipids VLPL mg/dL
VLDL Apo-B100 VLAB mg/dL
VLDL Particle Number VLPN nmol/L
IDL Triglycerides mg/dL
IDL Cholesterol IDCH mg/dL
IDL Free Cholesterol IDFC mg/dL
IDL Phospholipids IDPL mg/dL
IDL Apo-B100 IDAB mg/dL
IDL Particle Number IDPN nmol/L
LDL Triglycerides mg/dL
LDL Cholesterol LDCH mg/dL
LDL Free Cholesterol LDFC mg/dL
LDL Phospholipids LDPL mg/dL
LDL Apo-B100 LDAB mg/dL
LDL Particle Number LDPN nmol/L
HDL Triglycerides mg/dL
HDL Cholesterol HDCH mg/dL
HDL Free Cholesterol HDFC mg/dL
HDL Phospholipids HDPL mg/dL
HDL Apo-A1 HDA1 mg/dL
HDL Apo-A2 HDA2 mg/dL
VLDL-1 Triglycerides V1TG mg/dL
VLDL-1 Cholesterol V1CH mg/dL
VLDL-1 Free Cholesterol V1FC mg/dL
VLDL-1 Phospholipids V1PL mg/dL
VLDL-2 Triglycerides V2TG mg/dL
VLDL-2 Cholesterol V2CH mg/dL
VLDL-2 Free Cholesterol V2FC mg/dL
VLDL-2 Phospholipids V2PL mg/dL
VLDL-3 Triglycerides V3TG mg/dL
VLDL-3 Cholesterol V3CH mg/dL
VLDL-3 Free Cholesterol V3FC mg/dL
VLDL-3 Phospholipids V3PL mg/dL
VLDL-4 Triglycerides V4TG mg/dL
VLDL-4 Cholesterol V4CH mg/dL
VLDL-4 Free Cholesterol V4FC mg/dL
VLDL-4 Phospholipids V4PL mg/dL
VLDL-5 Triglycerides V5TG mg/dL

VLDL-5 Cholesterol V5CH mg/dL
VLDL-5 Free Cholesterol V5FC mg/dL
VLDL-5 Phospholipids V5PL mg/dL
VLDL-6 Triglycerides V6TG mg/dL
VLDL-6 Cholesterol V6CH mg/dL
VLDL-6 Free Cholesterol V6FC mg/dL
VLDL-6 Phospholipids V6PL mg/dL
LDL-1 Triglycerides L1TG mg/dL
LDL-1 Cholesterol L1CH mg/dL
LDL-1 Free Cholesterol L1FC mg/dL
LDL-1 Phospholipids L1PL mg/dL
LDL-1 Apo-B100 L1AB mg/dL
LDL-1 Particle Number L1PN nmol/L
LDL-2 Triglycerides L2TG mg/dL
LDL-2 Cholesterol L2CH mg/dL
LDL-2 Free Cholesterol L2FC mg/dL
LDL-2 Phospholipids L2PL mg/dL
LDL-2 Apo-B100 L2AB mg/dL
LDL-2 Particle Number L2PN nmol/L
LDL-3 Triglycerides L3TG mg/dL
LDL-3 Cholesterol L3CH mg/dL
LDL-3 Free Cholesterol L3FC mg/dL
LDL-3 Phospholipids L3PL mg/dL
LDL-3 Apo-B100 L3AB mg/dL
LDL-3 Particle Number L3PN nmol/L
LDL-4 Triglycerides L4TG mg/dL
LDL-4 Cholesterol L4CH mg/dL
LDL-4 Free Cholesterol L4FC mg/dL
LDL-4 Phospholipids L4PL mg/dL
LDL-4 Apo-B100 L4AB mg/dL
LDL-4 Particle Number L4PN nmol/L
LDL-5 Triglycerides L5TG mg/dL
LDL-5 Cholesterol L5CH mg/dL
LDL-5 Free Cholesterol L5FC mg/dL
LDL-5 Phospholipids L5PL mg/dL
LDL-5 Apo-B100 L5AB mg/dL
LDL-5 Particle Number L5PN nmol/L
LDL-6 Cholesterol L6CH mg/dL
LDL-6 Triglycerides L6TG mg/dL
LDL-6 Free Cholesterol L6FC mg/dL
LDL-6 Phospholipids L6PL mg/dL
LDL-6 Apo-B100 L6AB mg/dL
LDL-6 Particle Number L6PN nmol/L
HDL-1 Triglycerides H1TG mg/dL
HDL-1 Cholesterol H1CH mg/dL
HDL-1 Free Cholesterol H1FC mg/dL

HDL-1 Phospholipids H1PL mg/dL
HDL-1 Apo-A1 H1A1 mg/dL
HDL-1 Apo-A2 H1A2 mg/dL
HDL-2 Triglycerides H2TG mg/dL
HDL-2 Cholesterol H2CH mg/dL
HDL-2 Free Cholesterol H2FC mg/dL
HDL-2 Phospholipids H2PL mg/dL
HDL-2 Apo-A1 H2A1 mg/dL
HDL-2 Apo-A2 H2A2 mg/dL
HDL-3 Triglycerides H3TG mg/dL
HDL-3 Cholesterol H3CH mg/dL
HDL-3 Free Cholesterol H3FC mg/dL
HDL-3 Phospholipids H3PL mg/dL
HDL-3 Apo-A1 H3A1 mg/dL
HDL-3 Apo-A2 H3A2 mg/dL
HDL-4 Triglycerides H4TG mg/dL
HDL-4 Cholesterol H4CH mg/dL
HDL-4 Free Cholesterol H4FC mg/dL
HDL-4 Phospholipids H4PL mg/dL
HDL-4 Apo-A1 H4A1 mg/dL
HDL-4 Apo-A2 H4A2 mg/dL

Other Abbreviations:

ACS acute coronary syndromes
MI myocardial infarction
EMI elevation myocardial infarction
NSTEMI non-ST-elevation myocardial infarction
STEMI ST-elevation MI
NMR nuclear magnetic resonance spectroscopy
CV cardiovascular
CVD cardiovascular disease
ACD Atherosclerotic cardiovascular disease
CAD coronary artery disease
IHD ischaemic heart disease
TSP sodium 3-(trimethylsilyl)-[2,2,3,3-d_4]-1-propionate
Ala alanine
Crn creatinine
Glut glutamine
Gly glycine
His histidine
i-Leu isoleucine
Phe phenylalanine
Tyr tyrosine
Val valine
Ac acetic acid

Cit citric acid
For formic acid
Lac lactic acid
Pyr pyruvic acid
Gluc glucose
s-LDL small-dense Low Density Lipoproteins
l-LDL large Low Density Lipoproteins
m-LDL medium Low Density Lipoproteins
PN particle numbers
LDL-C LDL cholesterol (LDCH)
HDL-C HDL cholesterol (HDCH)
